# MicroRNA-30a contributes to pre-eclampsia through regulating the proliferation, apoptosis, and angiogenesis modulation potential of mesenchymal stem cells by targeting AVEN

**DOI:** 10.1080/21655979.2022.2054909

**Published:** 2022-03-24

**Authors:** Fangle Gu, Dan Lu, Liying Zhang

**Affiliations:** Department of Obstetrics, Clinical Medical College of Yangzhou University, Northern Jiangsu People’s Hospital, Yangzhou China

**Keywords:** MiR-30a, AVEN, HUVECs, PE, dMSCs

## Abstract

Pre-eclampsia (PE) is a pregnancy-associated disease related to an unprecedented hypertension attack. Mesenchymal stem cells (MSCs) play a crucial role in PE pathology. . Our research was designed to illustrate the functions of microRNA-30a (miR-30a) in proliferation, apoptosis, and the potential of regulating angiogenesis in MSCs, and to analyze its potential molecular mechanisms. TargetScan software and the luciferase reporter assay were used to forecast and verify the relationship between miR-30a and AVEN. MiR-30a and AVEN expression in the decidual tissue and decidua (d)MSCs of healthy pregnant women and PE patients were assessed using quantitative reverse transcription-polymerase chain reaction (qRT-PCR). Cell proliferation, 3-(4,5-dimethylthiazol-2-yl)-2,5-diphenyl-2 H-tetrazolium bromide (MTT), flow cytometry, and transwell assays were used to evaluate cell proliferation, growth, the cell cycle, apoptosis, and migration. Furthermore, the tube formation ability was evaluated using the human umbilical vein endothelial cell (HUVEC) tube formation assay. AVEN is the target gene of miR-30a. MiR-30a was upregulated in decidual tissues and dMSCs of PE patients. However, AVEN was weakly expressed, and AVEN expression was negatively related to miR-30a levels in decidual tissues and dMSCs of PE patients. Compared to the mimic control group, upregulation of miR-30a inhibited dMSC proliferation and cell growth, promoted G0/G1 phase arrest, and induced apoptosis. Furthermore, the miR-30a mimic transfected dMSC culture supernatant suppressed HTR-8/SVneo cell migration ability and HUVEC tube formation ability. However, AVEN reversed these changes. In conclusion, miR-30a/AVEN may serve as a new axis for PE treatment.

## Graphical abstract

**Figure:**
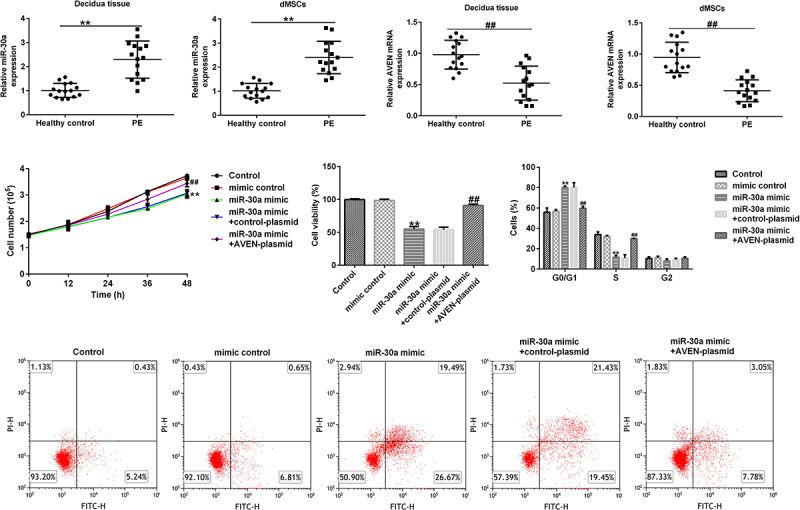

## Introduction

Pre-eclampsia (PE) is a pregnancy-related disease characterized by new-onset hypertension and proteinuria at ≥20 weeks of gestation [1–3]. After 20 weeks of gestation, the patient experiences new hypertension and proteinuria, as well as multiple organ dysfunction, including the liver, kidney, and brain [1,2,4]. PE is the principal cause of morbidity and death in pregnant women and newborns [3]. According to previous studies, insufficient angiogenesis may be one of the principal reasons for this [5]. Therefore, a good equilibrium between pro-angiogenesis and anti-angiogenesis is essential for the survival of maternal-fetal interface cells, which determines whether pregnancy can be successful [6,7].

Mesenchymal stem cells (MSCs) are multifunctional stem cells derived from various tissues, including the decidua, umbilical cord, and bone marrow [8]. MSCs are characterized by continuous self-renewal, expansion, and multidirectional differentiation into other cells [9,10]. Previous studies have shown that MSCs play a key role in immune regulation and angiogenesis [11–13]. MSCs can secrete a variety of biologically active molecules, such as vascular endothelial growth factor (VEGF), interleukin-6 (IL-6), and monocyte chemoattractant protein-1 (MCP-1) [12,14,15]. An important source of MSCs is the maternal-fetal interface [16–18]. It has been reported that MSCs at the maternal-fetal interface can ensure successful pregnancy [11].

MicroRNAs (miRNAs) are a type of small non-coding RNA, with a length of approximately 20–22 nucleotides, which differ from messenger RNA (mRNA) transcription proteins. MiRNAs do not encrypt proteins and restrain the expression of target genes [19–21]. Increasing evidence has shown that miRNAs participate in the development and metastasis of various types of cancers [22,23]. MiRNAs can also serve as biomarkers for disease diagnosis [24]. In addition, a previous study showed that there is a divergence in the expression of different miRNAs in the MSCs and maternal plasma of PE patients [25–29]. MiR-30a, a member of the miR-30 family, was shown to contain five different types of miRNAs, namely, miR-30a, miR-30b, miR-30c, miR-30d, and miR-30e [30,31]. According to previous studies, miR-30a acts as a biomarker for the development of various cancers [32,33]. Furthermore, miR-30a plays a key role in other diseases such as hepatic fibrosis, migraine, and endometritis. MiR-30a was over-expressed in decidua-derived MSCs (dMSCs) from patients with PE [34]. MiR-30a is also involved in the differentiation of osteoblasts and osteocytes of MSCs [35,36]. Niu et al. demonstrated that miR-30a-3p was upregulated in the placenta of patients with PE, and miR-30a-3p may affect the apoptosis and invasion of trophoblast cells by inhibiting insulin-like growth factor-1 (IGF-1 expression [37]. However, the various biological effects of miR-30a on MSCs have not yet been completely elucidated.

AVEN is an apoptosis inhibitor that exerts an inhibitory effect on B-cell lymphoma-X long (Bcl-xL) and apoptosis protease activator-1 (Apaf-1) [38,39]. Han et al. indicated that AVEN helps increase cancer cell chemotherapy drugs resistance [40]. Long et al. showed that AVEN participates in diabetic nephropathy development [41]. However, the expression and role of AVEN in PE remain unclear.

Through bioinformatics analysis, we found that AVEN may be a potential target gene of miR-30a. Thus, we hypothesized that miR-30a contributes to pre-eclampsia through regulating the proliferation, apoptosis, and angiogenesis modulation potential of MSCs by targeting AVEN. Therefore, this study explored the functions of miR-30a in proliferation, apoptosis, and the potential of regulating angiogenesis in MSCs, and analyzed its potential molecular mechanisms.

## Materials and methods

### Clinical samples

Human decidua tissue from patients with PE (PE: n = 15; gestational age at delivery 34–37 weeks) and age-matched uncomplicated pregnant women (healthy control: n = 15; gestational age at delivery 37–41 weeks) were obtained aseptically during cesarean section at the Clinical Medical College of Yangzhou University, Northern Jiangsu People’s Hospital. This study was approved by the ethics committee of the Clinical Medical College of Yangzhou University, Northern Jiangsu People’s Hospital. Written consent was obtained from the women before the surgery.

## Cells acquired and culture

Human umbilical vein endothelial cells (HUVECs) and HTR-8/SVneo cells were obtained from the ATCC. HTR-8/SVneo cells are immortalized human trophoblast cell systems. The two cell lines were cultured in RPMI-1640 medium (Gibco, NY, USA) with 10% fetal bovine serum (FBS) (Gibco) and cultured at 37°C in a 5% CO_2_ incubator.

## Decidual mesenchymal stem cells (dMSCs) isolation and culture

dMSCs were separated from the decidual tissues of healthy pregnant women and patients with PE [42]. First, the decidual tissue was washed several times with 1 × PBS (Gibco), then the machine was used to break the decidual tissue, incubate it in the enzyme mixture, and stir it gently at 37°C for 1 h. The enzyme mixture was washed with 1 × PBS and with Dulbecco’s Modified Eagle Medium/F12 (DMEM/F12) medium (Gibco). Finally, the cells were resuspended in fresh DMEM/F12 medium containing 20% FBS+1% antibiotics. The cells were cultivated in a 37°C, 5% CO_2_ incubator. After 2 d, the small digestive residues were removed and the culture was continued. Trypsin/EDTA (0.25%, Gibco) was used to separate the cells when a large number of colonies were observed, and transfer them to a new culture plate containing 10% FBS. After passage 2 to 4, flow cytometry (FCM) was used to detect the specific phenotypic surface antigens of MSCs.

## Cell transfection

dMSCs (5 × 10^4^ cells/well) were induced by the inhibitor control, miR-30a inhibitor, mimic control, miR-30a mimic, control-plasmid, or AVEN-plasmid using Lipofectamine® 3000 reagent (Thermo) for 48 h at 37°C, following the manufacturer’s instructions. Cell transfection efficiency was determined using quantitative reverse transcription-polymerase chain reaction (qRT-PCR).

Quantitative reverse transcription-polymerase chain reaction **(qRT-PCR) assay**

Total RNA was collected using TRIzol reagent (TaKara, Shiga, Japan), according to the manufacturer’s protocol. RNA was detected using NanoDrop (Thermo Scientific, USA). When RNA collection was successful, RNA was transformed into complementary DNA (cDNA) using the PrimeScript RT Reagent Kit (TaKara). Subsequently, qPCR was performed using the SYBR Green PCR Kit (Vazyme, Nanjing, Jiangsu), according to the protocol of the reference. Gene expression was calculated using the 2^−ΔΔCt^ formula [43].

## Cell proliferation assay

Cell proliferation of dMSCs was evaluated using plating appropriate numbers in 12-well plates (Corning, Lowell, MA, USA), cultivating for different times (12, 24, 36, and 48 h), followed by harvesting and counting [44].

## 3-(4,5-dimethylthiazol-2-yl)-2,5-diphenyl-2 H-tetrazolium bromide (MTT) assay

Cell viability was determined usiing MTT assay [45]. After treatment, 20 µL 3-(4,5-dimethylthiazol-2-yl)-2,5-diphenyl-2 H-tetrazolium bromide (MTT, 5 mg/mL, Sigma) was added to the wells, and the cells were continuously incubated for an additional 4 h. Next, the culture medium was dislodged, and 200 µL dimethyl sulfoxide (DMSO) was added to the wells. The optical density (OD) at a wavelength of 570 nm was measured using a microplate reader.

## Flow cytometry (FCM) assay

For the cell cycle assay, after the cells were transfected for 48 h, they were fixed in pre-cooled 70% ethanol and incubated overnight at 4°C. Next, the cells were washed twice with 1 × PBS and then with 50 µg/mL propidium iodide (PI) (BD Bioscience), and 20 µg/mL RNase A was added to the cells, followed by cultivation at room temperature for 30 min, and detection using FAC [46]. For the cell apoptosis assay, transfected cells were assessed using the Annexin V/propidium iodide (PI) Apoptosis Detection Kit [47]. Briefly, cells were washed twice with pre-cooled 1 × PBS before collecting cells, and then a cell suspension of 1 × 10^6^ cells/mL was prepared using FITC-binding buffer. An amount of 100 μL of the cell suspension was added to EP tubes. Subsequently, an appropriate amount of Annexin V-FITC and PI was added to the cells according to standard procedures. The cells were mixed gently and cultivated for 20 min at room temperature in the dark. Annexin V-FITC and PI fluorescence were detected using a BD FACSCalibur flow cytometer (BD Technologies).

## Western blot assay

Protein expression was detected by western blot assay in this study [48]. Cells were lysed using RIPA buffer for 30 min on ice (Solarbio, Beijing, China). Protein consistency was obtained using NanoDrop. Proteins were resolved using 12% SDS-PAGE and transferred onto PVDF membranes. The membranes were blocked with 5% skimmed milk for 2 h to avoid nonspecific binding, and then incubated with primary antibodies against anti-AVEN or anti-GAPDH (1:1000, Abcam) at 4°C overnight. Next, they were washed three times and cultivated with a secondary antibody for 2 h. The protein signals were assessed using the ECL method (Applygen Technologies, Inc.).

## Dual luciferase reporter analysis

To verify the binding sites of miR-30a and AVEN, dual luciferase reporter analysis was performed [49]. We then constructed AVEN-WT and AVEN-MUT 3’-UTR luciferase reporter gene plasmids. 293 T cells were transfected with Renilla luciferase, luciferase reporter gene plasmids, and miR-30a mimic or mimic control for 48 h. Luciferase activity was assessed using a dual-luciferase reporter assay system (Promega), following the manufacturer’s instructions.

## HUVEC tube formation assay

We mixed Matrigel and serum-free medium at a ratio of 1:1, and then added the diluted Matrigel to a 24-well plate and allowed it to solidify. dMSCs were exposed to the mimic control, miR-30a mimic, control-plasmid, or AVEN-plasmid for 48 h, and the culture supernatant was collected from each group of dMSCs. HUVEC cells (1 × 10^5^) with an equal volume of the cell supernatant collected previously were inoculated on the coagulated Matrigel and incubated for 8 h. Five fields were randomly selected for analysis using Image Pro Plus software. The mean number of complete tubes formed by HUVECs was counted [50].

## HTR-8/SVneo migration assay

For the migration assay [50], a transwell chamber (8-μm pore size, Millipore) was placed in a 24-well plate. dMSCs were transfected with the mimic control, miR-30a mimic, control-plasmid, or AVEN-plasmid for 48 h, and the culture supernatant was collected from each group of dMSCs. HTR-8/SVneo cells (1 × 10^5^) were resuspended with 500 μL DMEM/F12 medium with 10% FBS, and the cells were seeded in the upper chamber. Subsequently, 500 μL of dMSC culture supernatant was added to the bottom chamber. The 24-well plate was plated at 37°C in a 5% CO_2_ incubator for 8 h. Next, the cells were fastened with 4% polyoxymethylene (Solarbio, Beijing, China) and stained with 0.1% crystal violet solution (Solarbio) for 20 min at room temperature. Cotton swabs were used to remove cells that had not migrated. The stochastically selected area was photographed using an optical microscope to count the number of migrated cells.

## Statistical analysis

SPSS11 software was used for the statistical analysis. The statistical significance of the difference between groups was determined using Student’s t-test or one-way Analysis of Variance (ANOVA). Data are shown as the mean ± standard deviation (SD) from three independent experiments. Statistical significance was set at *p* < 0.05.

## Results

### MiR-30a was upregulated in the decidua tissue and dMSCs of patients with PE

**Figure 1.**
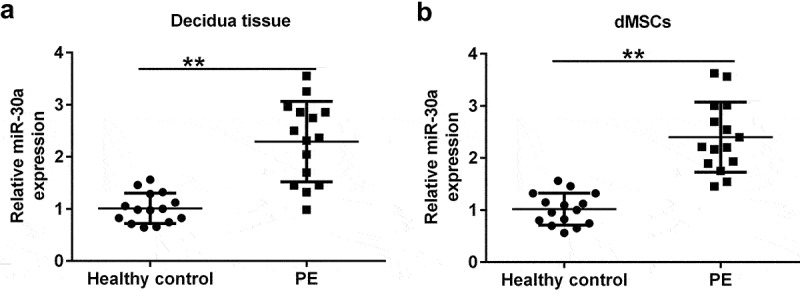
**MiR-30a levels were increased in the decidua tissue and decidua MSCs (dMSCs) of patients with pre-eclampsia (PE)** Quantitative reverse transcription-polymerase chain reaction (qRT-PCR) analysis of miR-30a levels in decidua tissue (a) and dMSCs (b) from healthy pregnancies and patients with PE. **P < 0.01.

First, decidua tissue and dMSCs were acquired from 15 healthy pregnancies and 15 patients with PE. qRT-PCR analysis was performed to assess miR-30a expression in decidua tissue and dMSCs. Our results showed that compared to healthy pregnancies, miR-30a was highly expressed in the decidua tissue (Figure 1a) and in dMSCs (Figure 1b) of patients with PE.

## AVEN was a direct target of miR-30a

**Figure 2.**
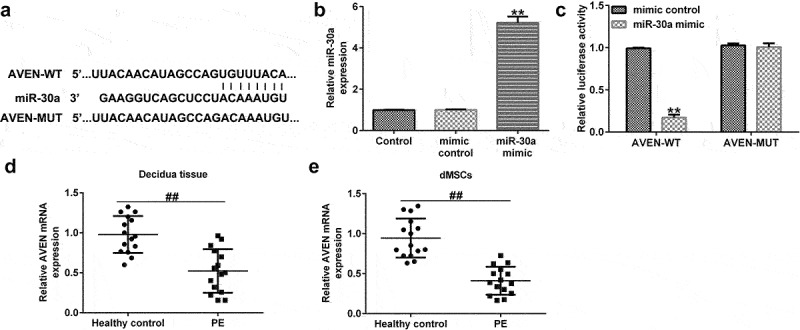
**AVEN directly targeted miR-30a** (a). Binding site between miR-30a and AVEN was predicted using TargetScan. (b) Determination of miR-30a in 293 T cells after mimic control or miR-30a mimic transfection. (c). Dual-luciferase assay was performed to verify the relationship. Quantitative reverse transcription-polymerase chain reaction (qRT-PCR) analysis determined AVEN expression in the decidua tissue (d) and decidua MSCs (dMSCs) (e) of healthy pregnancies and patients with PE. **P < 0.01 vs. mimic control group; ^##^P < 0.01 vs. Healthy control.

To predict the downstream target gene of miR-30a, we used TargetScan analysis software to predict target genes, and a dual-luciferase assay was used to confirm the correlation between miR-30a and the target gene. In this study, the TargetScan assay predicted that there was a binding site for miR-30a and AVEN (Figure 2a). It was then confirmed that upregulation of miR-30a significantly enhanced miR-30a expression in 293 T cells (Figure 2b). 293 T cells were treated with AVEN-WT or AVEN-MUT luciferase reporter plasmid, miR-30a mimic, and Renilla luciferase reporter plasmid for 48 h. Dual-luciferase reporter analysis suggested that the miR-30a mimic suppressed AVEN-WT activity. However, the activity of AVEN-MUT showed no obvious changes (Figure 2c). Next, qRT-PCR assay demonstrated that AVEN was downregulated in the decidua tissue (Figure 2d) and dMSCs (Figure 2e) of patients with PE compared to the healthy control group.

## MiR-30a negatively regulated AVEN expression in dMSCs

**Figure 3.**
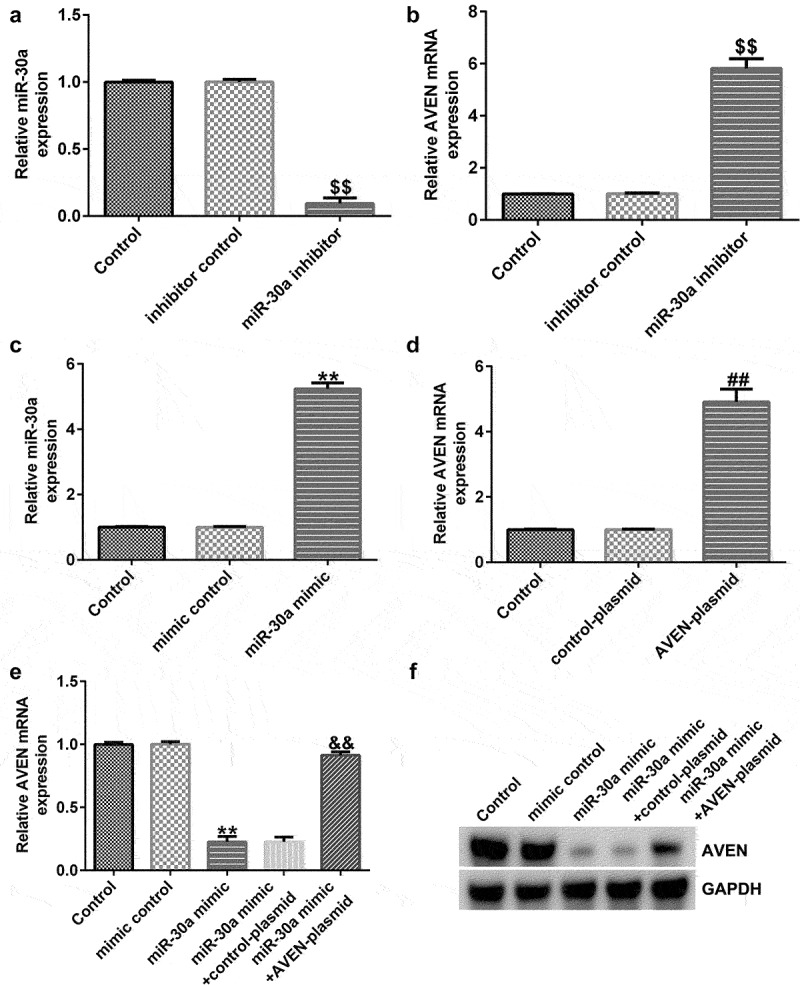
**MiR-30a negatively regulated AVEN expression**(a). Quantitative reverse transcription-polymerase chain reaction (qRT-PCR) analysis of miR-30a levels in inhibitor control or miR-30a inhibitor transfected decidua MSCs (dMSCs). (b). Detection of AVEN expression in inhibitor control or miR-30a inhibitor transfected dMSCs using qRT-PCR. (c). qRT-PCR analysis of miR-30a levels in mimic control or miR-30a mimic transfected dMSCs. (d). Detection of AVEN expression in control-plasmid or AVEN-plasmid transfected dMSCs using qRT-PCR. Expression of AVEN in the mimic control, miR-30a mimic, control-plasmid, or AVEN-plasmid transfected dMSCs using qRT-PCR (e) and western blot assay (f). ^$$^P < 0.01 vs. inhibitor control group; **P < 0.01 vs. mimic control group; ^##^P < 0.01 vs. control-plasmid group; ^&&^P < 0.01 vs. miR-30a mimic+control-plasmid group.

To further illustrate the effects of miR-30a on AVEN expression in dMSCs, inhibitor control, miR-30a inhibitor, mimic control or miR-30a mimic was transfected into dMSCs for 48 h. As shown in Figure 3a, miR-30a inhibitor significantly reduced miR-30a expression compared to the inhibitor control group. Meanwhile, compared with the inhibitor control group, the mRNA level of AVEN was significantly enhanced in miR-30a inhibitor transfected dMSCs (Figure 3b). As presented in Figure 3c, upregulation of miR-30a improved miR-30a expression compared to the mimic control group. In addition, after control-plasmid or AVEN-plasmid transfection, AVEN expression was increased (Figure 3d). Further qRT-PCR and western blot assays indicated that compared to the mimic control group, the miR-30a mimic reduced AVEN levels, and this decrease was reversed by the AVEN-plasmid (Figure 3 e and f).

## MiR-30a suppressed dMSCs proliferation and induced cell cycle arrest and cell apoptosis

**Figure 4.**
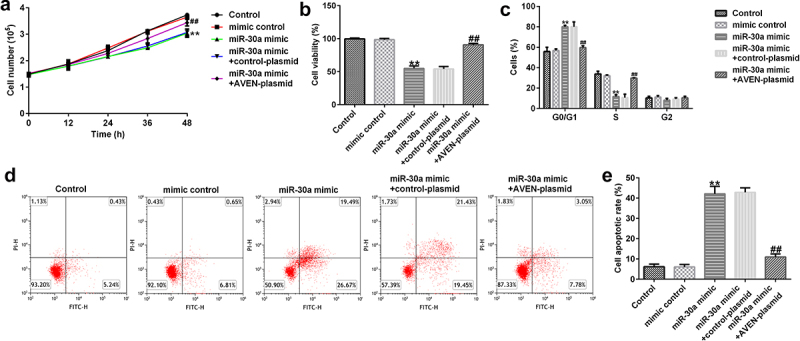
**MiR-30a suppressed decidua MSCs (dMSCs) proliferation, induced cell cycle arrest, and apoptosis by targeting AVEN** (a) Cell proliferation was assessed using cell proliferation assay. (b). The growth of dMSCs was determined using the 3-(4,5-dimethylthiazol-2-yl)-2,5-diphenyl-2 H-tetrazolium bromide (MTT) assay. (c). Flow cytometry (FCM) was used to analyze cell numbers in different stages of cell division and the cell cycle data was shown using Graphpad 6.0. (d). FCM analysis of apoptotic cells. (e). Cell apoptosis rate was shown using Graphpad 6.0. **P < 0.01 vs. mimic control group; ^##^P < 0.01 vs. miR-30a mimic+control-plasmid group.

Subsequently, the role of miR-30a in dMSC viability was investigated. The mimic control, miR-30a mimic, control-plasmid, or AVEN-plasmid were transfected into dMSCs for 12, 24, 36, or 48 h. MTT assay revealed that compared to the mimic control group, the miR-30a mimic inhibited cell proliferation and viability, while this inhibition was reversed by the AVEN-plasmid (Figure 4 a and b). Moreover, FCM analysis demonstrated that the miR-30a mimic promoted cell cycle arrest in the G0/G1 phase (Figure 4c) and induced apoptosis (Figure 4 d and e). However, these findings were eliminated by the AVEN plasmid.

## MiR-30a overexpressed dMSCs regulated angiogenesis by targeting AVEN

**Figure 5.**
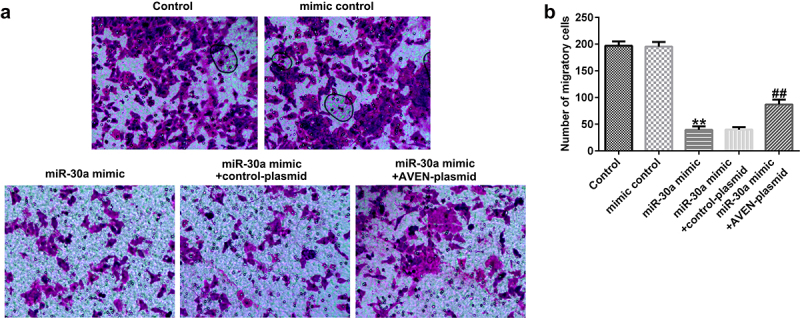
**Decidua MSCs (dMSCs) overexpressed miR-30a suppressed HTR-8/SVneo migration**. (a). HTR-8/SVneo cells migration was evaluated using transwell assay. (b). Number of migratory HTR-8/SVneo cells were presented. **P < 0.01 vs. mimic control group; ^##^P < 0.01 vs. miR-30a mimic+control-plasmid group.

**Figure 6.**
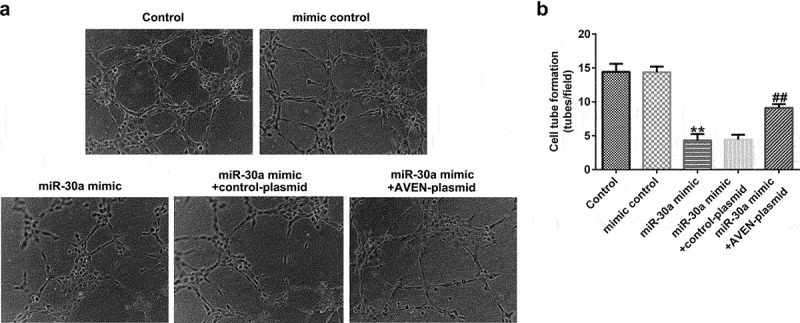
**Decidua MSCs (dMSCs) overexpressed miR-30a suppressed human umbilical vein endothelial cell (HUVEC) tube formation**.(a). Determination of HUVEC tube formation ability. (b). HUVEC tube formation was quantified. **P < 0.01 vs. mimic control group; ^##^P < 0.01 vs. miR-30a mimic+control-plasmid group.

Finally, we explored the effects of miR-30a overexpressed dMSCs on HTR-8/SVneo cell migration and HUVEC tube formation. We performed a transwell assay to explore HTR-8/SVneo cell migration. Compared to the mimic control, miR-30a overexpressed dMSC culture supernatant significantly inhibited HTR-8/SVneo migration ability. However, the AVEN-plasmid reversed the inhibitory effect (Figure 5 a and b). The HUVEC tube formation assay demonstrated that the miR-30a mimic transfected dMSC culture supernatant significantly suppressed HUVEC tube formation, and this change was reversed by the AVEN-plasmid (Figure 6 a and b).

## Discussion

PE is a relatively common pregnancy disorder that can threaten the survival of both mother and baby [1–3]. Some evidence suggests that PE is a systemic vascular disease that may suggest later cardiovascular disease in the mother [51,52]. Previous studies have highlighted the role of disturbances in the balance of angiogenesis as one of the main features of this disease [53]. MSCs are capable of self-renewal and have the potential to differentiate into mesenchymal and non-mesenchymal tissues, thus, they have great potential to treat various diseases [54]. The role of MSCs in angiogenesis and their medical application value have been extensively studied [55]. Furthermore, it has been recently reported that placenta-derived MSCs contribute to vascular maturation and stabilization [56]. In addition, MSCs can secrete factors that promote the formation of endogenous blood vessels and nerves [57]. Currently, the roles of dMSCs in PE remain largely unknown. Multiple studies have illustrated that miRNAs are related to pathogenesis of PE [58–61]. Hu et al. showed that miR-30a was upregulated in the umbilical cord tissue and dMSCs of patients with PE. Our results are consistent with previous research findings [61] that miR-30a is highly expressed in decidual tissue and dMSCs from patients with PE. A previous study indicated that miR-16 and miR-136 are also upregulated in dMSCs [59,60].

Next, a dual luciferase reporter gene assay confirmed that AVEN was a direct target of miR-30a. In PE patients, AVEN was downregulated and negatively correlated with miR-30a level.

In addition, we observed that miR-30a suppressed dMSC proliferation and induced apoptosis through down-regulating AVEN expression. Similar to our results, Ji et al. revealed that miR-136 suppresses MSC ability and induces cell apoptosis [59]. A report by Wang et al. showed that miR-16 overexpression suppressed cell growth and resulted in the accumulation of dMSCs in the G0/G1 phase [60]. In our study, miR-30a induced cell cycle arrest in the G0/G1 phase. These changes were reversed by the application of AVEN.

The progression of severe PE is highly associated with a decline in trophoblast cell invasion ability and is an obstacle to uterine spiral arteriole remodeling [62]. In this study, we found that miR-30a overexpressed dMSC culture supernatant suppressed HTR-8/SVneo cell migration ability by targeting AVEN. Moreover, the findings revealed that miR-30a overexpressed dMSC culture supernatant significantly reduced HUVEC tube formation ability, indicating that miR-30a significantly reduced the angiogenesis modulation potential of MSCs.

## Conclusion

The findings revealed for the first time that miRNA-30a regulates the viability, apoptosis, and angiogenesis-regulating potential of MSCs by targeting AVEN and accelerates the occurrence and progression of eclampsia.

## Availability of materials and data statement

The datasets used and/or analyzed during the present study are available from the corresponding author on reasonable request.
